# Comparison of Two brands of Methylphenidate (Stimdate^®^ vs. Ritalin^®^) in Children and Adolescents with Attention Deficit Hyperactivity Disorder: A Double-Blind, Randomized Clinical Trial

**Published:** 2012

**Authors:** Naser Khodadust, Amir-Hossein Jalali, Masoud Ahmadzad-Asl, Noushin Khademolreza, Elham Shirazi

**Affiliations:** 1 Psychiatrist, Mental Health Research Center, Tehran Psychiatric Institute, Tehran University of Medical Sciences and Health Services, Tehran, Iran.; 2 Assistant Professor of Psychiatry, Mental Health Research Center, Tehran Psychiatric Institute, Tehran University of Medical Sciences and Health Services, Tehran, Iran.; 3 Resident of Psychiatry, Mental Health Research Center, Tehran Psychiatric Institute, Tehran University of Medical Sciences and Health Services, Tehran, Iran

**Keywords:** ADHD, Ritalin, Side effects, Stimdate, Therapeutic effect

## Abstract

**Objective:** To compare the effectiveness and safety of the methylphenidate produced in Iran (Stimdate®) with its original brand (Ritalin®) in children with Attention deficit hyperactivity disorder (ADHD).

**Methods:** In this double-blinded randomized clinical trial, 30 patients with ADHD who were 6 to 16 years old, were divided into two groups: 15 in Stimdate® and 15 in Ritalin® group. The two groups were compared for side effects profile, Conner’s Parent’s Rating Scale-Persion version (CPRS-R), Child Symptom Inventory-4 (CSI-4), Clinical Global Impressions (CGI), and Children’s Global Assessment Scale (CGAS), at baseline and at the 4^th^ and 6^th^ weeks.

**Results: **The subjects showed significant decreases in the CPRS-Rand CSI-4 scores and significant increase of CGAS scores during the follow-up, but there were no significant difference between Stimdate® and Ritalin® group, regarding the pattern of changes observed. The mean therapeutic dose and the number of side effects were not significantly different between the two studied groups.

**Conclusions:** Both Stimdate® and Ritalin® had comparable clinical efficacy and safety in children with ADHD.

## Introduction

Attention deficit hyperactivity disorder (DHD) is one of the most prevalent diagnoses in pediatric psychiatry worldwide, with the rate of 5-10%, which spans preschool to adult years (1-3). The disorder has severe dysfunctional symptoms which affect personal, social, and educational aspects of patients’ life and therefore necessitates intense treatment (4,5). ADHD is known to have heterogeneous etiological pathways such as genetic, neurologic, environmental, and other factors that influence early neurological and brain development. Reported etiologies include prenatal stress, low birth weight, prenatal smoking and alcohol use, obstetric complications, head injury, and epilepsy (6). A strong body of evidence suggests that central nervous system stimulants (e.g., methylphenidate; MPH) are the most effective therapies available in controlling ADHD symptoms throughout the day, thus are proposed as its first line therapeutic medications (7). The first report of stimulant use to treat ADHD was in 1937(8). Seventy-five percent of children respond to the first stimulant medication trial (9-12). The data also consistently indicate that MPH is more officious than no pharmacological interventions.

MPH, a psycho stimulant and a derivative of amphetamine, is a controlled drug that is recommended for use as part of treatment programs for children with a confirmed diagnosis of ADHD (13). MPH, better known by its trade name Ritalin^®^ (manufactured by Novartis), has been used to treat ADHD since 1954. It is also noteworthy that there exist other pharmacological and non-pharmacological treatments for patients with ADHD such as bupropion, clonidine, guanfacine, moclobemide, selegiline, modafinil and atomoxetine as well as psychoeducation, psychotherapies, and family interventions (7, 9, 14,-21).

In developing countries like Iran, the original brand of the drug is not widely distributed and its price is beyond the purchasing power of most of patients. Furthermore, insurance companies do not usually cover the aforementioned imported drugs. 

There are some brand names of MPH in Iran, mostly Ritalin^®^ (the original brand of MPH) manufactured by Novartis Company and Stimdate^®^ (Local Brand of MPH) manufactured by Iranian Mehrdaru Company in Iran. As mentioned earlier, prescribing Stimdate® instead of Ritalin^®,^ in ADHD patients is somewhat an obligation. The ethnic, cultural, and economic diversities may play a role in medication efficiency, by affecting the adherence to specific types of medication brands, and therefore the rate of symptoms relief achieved by each drug (22).

In this study we aimed to compare the therapeutic effect of Ritalin® made by Novartis Company with Stimdate® made by Iranian Company of Mehrdaru in children and adolescent with ADHD in Iran. We designed this study to compare these two drugs in terms of their effect on ADHD symptoms, which would prepare us for future assessment of drug efficiency in community. 

## Materials and Methods

*Participants*

Thirty 6-16 years old children and adolescents who were diagnosed as having ADHD (combined type) by means of The Diagnostic and Statistical Manual of Mental Disorders-Fourth Edition (DSM-IV-TR) (4), Children Symptom Inventory-DSM-IV version (CSI-4), and clinical judgment of a child and adolescent psychiatrist and a senior resident of psychiatry, were recruited in the study according to convenient sampling method, from patients referred to the outpatient psychiatric clinics of Tehran University of Medical Sciences affiliated hospitals including Hazrat Rasul-Akram (S) hospital clinic, and clinic of Tehran Psychiatric Institute, Tehran, Iran. 

All participants have to met the following criteria to be included in the study: 1) being 6-16 years old; 2) meeting the DSM-IV^4^diagnostic criteria for ADHD; 3) No psychological or medical treatment received in the last 4 weeks before the study; 4)having informed written consent signed by parents for participating in the study; 5) not having co morbid conditions [please check to be correct] including conduct disorder, pervasive developmental disorder, mood disorders,, Tourette’s disorder, and psychotic disorders; and 6) the ability to comply with the study’s visits schedule. No monetary compensation was provided to the families for participation in the study. The exclusion criterion were the following: 1) the presence of clinically significant gastrointestinal problems, cardiovascular diseases, glaucoma, and seizure disorder, 2) suspicion or confirmation of substance abuse by patients or a family member; 3) presence of mental retardation according to educational history or, having an IQ score less than 70; 4) allergy to stimulants; and 5) having to receive any psychiatric or somatic medication (except Ritalin or Stimdate) during the study. 

Two patients were excluded from the study. The first was case No. 17 in Stimdate® group who was excluded because he fainted in the 3^rd^ week of treatment, and the second one was case No. 23 in Ritalin® group who experienced several side effects at the first week of treatment with Ritalin®.

*Intervention*

This study was a randomized double blind-controlled trial with active control, to compare the clinical efficacy of Stimdate^®^ and Ritalin^®^. 

Thirty patients were allocated to each group. We used sequentially numbered containers (SNCR) method for randomization. All of the containers were tamper proof, equal in weight, and similar in appearance. The first researcher allocated a series of container to patients with the code of “1” or “2”. The second researcher performed the assessments and was blinded to the groups. 

We used an active control (positive control) Stimdate® tablet were produced in the same shape, color, and weight similar of Ritalin®. We used the same containers for both groups.

Before entering the cases in the study, a complete physical exam during which the subject’s heart rate, blood pressure, and weight were measured, was performed. This exam was also repeated in the 4^th^ week to exclude any case with possible problems. The treatment protocols for both Ritalin® and Stimdate® groups were as follows: starting with 5 mg at morning and noon and weekly increments by 5mg in each dose, until reaching the maximum dose of 20mg at morning and noon in week four.

In case the weight of the child was less than 20 kg, the maximum daily dose would not exceed 30mg. The treatment dose for the 5^th^ and 6^th^ weeks was determined according to the best treatment response during the 1^st^ to 4^th^ weeks.

*Main measurements*

The Standard Persian version of the Conner’s Parents Rating Scale-Revised (CPRS-R) was used for the assessment of the severity of ADHD. The CPRS-R is used widely in measurement of the treatment efficacy and for outcome assessment purposes in ADHD children and adolescents (aged 3-17). The test has been reported to have the validity rate of 0.84 (23). The Child Symptom Inventory-DSM-IV version (CSI-4) (ADHD part) can be used for diagnostic purposes in clinical settings, and is also used to measure symptom severity by clinicians, teachers and parents (24, 25). The internal consistency, and reliability for CSI-4 has been reported to be 0.74-0.94 in the literature and it has acceptable criterion validity (25, 26). The CGI Scale (25) is used by clinicians, to rate the severity of the illness, its changes over time, and the efficacy of medications which are used in the treatment process, taking into account the patient’s clinical condition and the severity of the side effects, The CGAS (27,28). is used to measure the overall functional status and functional disturbances in children and adolescents. The CGI and CGAS have showed acceptable reliability and validity scores in different studies (29-31). 

CPRS-R (32) and drug side effects were assessed at baseline and by weekly telephone calls thereafter, in the subjects. CSI, CGI, and CGAS were completed at baseline and at the end of the 4^th^ and 6^th^ weeks of the study 

*Statistical analysis*

We used SPSS software for windows (Ver. 11.5) (SPSS Inc. Chicago, Ill) for data analysis. Descriptive analyses were generated for all parameters. Differences in CPRS-R, CSI, CGAS, and CGI parameters were calculated in each visit. Analyses of the efficiency, based on the differences in scores of the parameters, were compared between the Ritalin® and the Stimdate® groups, using paired t-test and repeated measures analysis. The differences of CPRS-Rscores in the 1^st^, 2^nd^, 3^rd^, 4^th^ and 6^th^ weeks, and CSI and CGAS scores, in the 4^th^ and 6^th^ weeks were calculated for each case. Since age is a confounding factor in the performance of most cognitive tests, we used the age of the participants as a covariate during analyses. All tests were two-sided with determining 0.05 as the level of significance.

## Results

The mean (SD) ages of the subjects were 9.2 (±0.5) and 8.33 (±0.5) years in Ritalin^®^ and Stimdate^®^ groups, respectively (P=0.21). The Ritalin^®^ group consisted of 12 males and 3 females; and the Stimdate^®^ group had 15 males (P= 0.22) (Table 1).

The mean (±SD) CPRS-R score was 50.33 (±2.7) for the Ritalin^®^ group and 55.7 (±1.9) for the Stimdate^®^ group, before treatment. The CPRS-R score showed significant decrease in both groups, from baseline to the 6^th^ week (P<0.01). There were no significant differences in the pattern of CPRS-R score’s reductions between the two treatment groups (Table 2).

**Table 1:** Baseline characteristics and treatment profile of subjects treated with Ritalin^® ^and Stimadate^®^

	Ritalin^®^ group	Stimdate^®^ group	P.
Age; years old, Mean±SD	9.2±0.5	8.3±0.5	N.S.†
Gender			N.S.
Male, n	12	15	
Female, n	3	0	
Ethnicity	All Persian	All Persian	N.S.
Final treatment dose; mg, Mean±SD	29.2±9.1	31.4±8.6	N.S.
Number of side effects n, Mean±SD	1.6±1.5	2.1±2.5	N.S.

†N.S.: Not Significant

**Figure 1:**
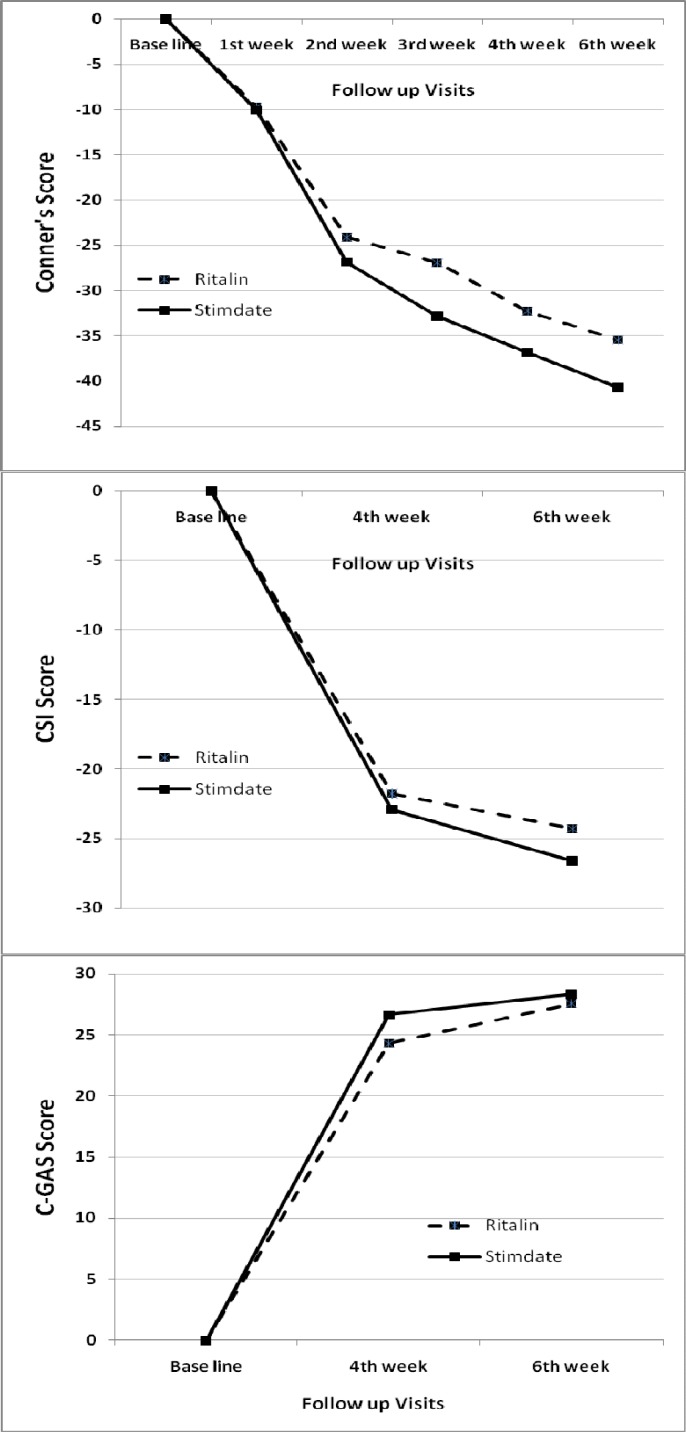
Changes in CPRS-R, CSI, and CGAS during follow up in Ritalin® and Stimdate® groups

Before treatment, the mean (SD) CSI scores were 34.8 (±1.6) and 37.6 (±1.5) , and CGAS values were 57 (±5.9) and 56.6 (±6.1), for the Ritalin^®^ and Stimdate^®^ groups, respectively (Table 1 and Fig. 1).The Ritalin^®^ and the Stimdate^®^ groups were similar according to their CPRS-R, CSI, and CGAS scores, before treatment (P > 0.05) (Table 2).

Mean (SD) values for CGI in the 4^th^ and the 6^th^ weeks after treatment were 1.57 (±0.2) and 1.62 (±0.3) for the Ritalin^®^ group and 1.64 (±0.2) and 1.33 (±0.2) for the Stimdate^®^ group, respectively (Figure 1). 

There were significant decreases in CPRS-R and CSI scores, and also significant increase in CGAS scores, in both groups during the follow-up period, but CGI showed no significant change during this period in any of the groups (Table 2 and Figure 1). However, there was no significant difference between the two groups in terms of the change patterns of the aforementioned parameters (Table 2 and Figure 1).

The mean (SD) of final treatment doses which were defined as the mean doses of drugs in the 4^th^ to 6^th^ weeks were 29.2 (±9.1) mg in the Ritalin^®^ group and 31.4 (±8.6) mg in the Stimdate® group (P = 0.59). The mean (SD) number of experienced side effects, was higher in Stimdate® group, in comparison to the Ritalin® group, but it was not statistically significant (2.13 (±2.5) vs. 1.6 (±1.5), respectively; P> 0.05).

**Table 2:** Measured parameters of ADHD subjects in Ritalin® and Stimdate® groups during follow up

	Ritalin^®^ group				Stimdate^®^ group				P §
	Week0	Week4	Week6	*P * †	Week0	Week4	Week6	*P* ‡	
CPRS-R Mean±SD	50.3±2.6	14.7±3.4	14.0±4.5	*0.02*	55.7±1.9	22.3±4.6	15.6±4.0	*0.02*	*N.S.*
CSI; Mean±SD	34.8±1.6	11.1±2.3	10.3±3.3	*<0.01*	37.6±1.5	15.8±2.7	10.9±2.4	*<0.01*	*N.S.*
CGI; Mean±SD		1.57±0.2	1.62±0.3	*N.S.* ║		1.6±0.2	1.3±0.2	*N.S.*	*N.S.*
CGAS; Mean±SD	57.0±1.6	83.8±2.1	85.2±2.7	*<0.05*	56.6±1.5	81.2±3.3	84.8±2.8	*<0.05*	*N.S.*

N.S.: Not Significant;

║: all P.values were calculated by repeated measures test,

†§: repeated measures test within groups changes in score in follow up period,

‡: test for differences between groups in pattern of changes during follow up.

## Discussion

ADHD is one of the most prevalent behavioral and psychiatric disorders among children worldwide (2). According to the high prevalence of ADHD among children and the need to provide patients with accessible and effective pharmacologic therapy, we performed this study to compare the effectiveness of methylphenidate produced in Iran (Stimdate^®^) with its original brand (Ritalin^®^). The two study groups were similar according to their age, sex, and intensity of disease (according to the CPRS-R, CSI, and CGAS scores) before treatment. Generally Stimdate^®^ showed more intense reduction in the CPRS-R, CSI, and CGAS scores. However, our finding proposes that the amount of this reduction was not statistically different from that of the Ritalin^®^ group.

Our study revealed that likewise baseline characteristics and treatment profile of the two groups, the measured efficacy parameters of ADHD subjects showed no significant differences between Stimdate^®^ and Ritalin^®^. In comparison to Ritalin^®^, considering the lower price of Stimdate^®^ ,and its broader distribution in our country, which provides a wide and easy accessibility to this medication, it seems that Stimdate^®^ can show even more efficiency in the community scales, during the treatment process of ADHD subjects in Iran, however this can be an issue that needs further and more comprehensive studies to be confirmed. Furthermore, the total number of side effects experienced by the subjects of the Stimdate^® ^group, was not significantly different from those of the Ritalin^®^ group, that can emphasize the comparable safety profile of both medications MPH in different forms of release (instant, extended, sustained, and so on) is the most common drug used for ADHD worldwide (33-36) and there is an emerging need to provide efficient medication supplies for children and adolescent with ADHD throughout the country.

In a similar study, Mohammadi et al. (37) Compared sixty children with ADHD treated with Stimdate^®^ and Ritalin^®^ and showed no significant differences between the two groups regarding Attention Deficit Hyperactivity Rating Scale (38). However, they did not report other parameters that we provided in our study such as the CPRS-R, CSI, and CGAS scores. While these two studies have similar results and methodologies, except regarding their sample sizes and measured parameters, we believe that our report would expand the body of evidence for similarities of these two brands.

Because of the short follow-up duration, and the small sample sizes, our study lacks to prepare generalizable data, therefore regarding the importance of prescribing an effective medication for ADHD patients, and the wide use of these brands in our country, we suggest further multi-center studies in this issue.

## Conclusion

We recommend clinicians to choose Ritalin® or Stimdate® according to the patient’s preferences, sustained accessibility, primary response to treatment, and possible side effects encountered in course of treatment. This means that none of these drugs have been proved to be superior to the other one.

## Authors' contributions

NKD participated in designing and manuscript preparation. NKR participated in allocation, and the initial and follow-up clinical assessments. AHJ re-evaluated the clinical data and revised the manuscript. MAA interpreted the clinical data, performed the statistical analysis and prepared the draft and revised the manuscript. ES conceived and designed the evaluation, re-analyzed the data, and revised and finalized the manuscript. All authors have read and have approved the final manuscript.

## Notes

**Declaration of interest:** None.

**Clinical Trial Registration-URL: **http://www.irct.ir. Unique identifier: IRCT201106306923N1.
